# Novel wearable and contactless monitoring devices to identify deteriorating patients in the clinical setting: a systematic review protocol

**DOI:** 10.1186/s13643-020-01370-1

**Published:** 2020-05-06

**Authors:** Peter Y. Chan, John McNeil, Tam Nguyen, Nicholas Ryan, Ingrid Hopper

**Affiliations:** 1 Department of Intensive Care Medicine, Box Hill Hospital, 8 Arnold Street, Box Hill, Victoria, 3128 Australia; 2 School of Public Health and Prevention Medicine, Monash University, Melbourne, Australia; 3 St Vincent’s Hospital, Melbourne, Victoria Australia

**Keywords:** Monitoring, Wearables, Contactless, Continuous, Vital signs, Deteriorating patient

## Abstract

**Background:**

With technological advances, there has been increasing interest in developing contactless and/or non-invasive wearable technologies that continuously monitor vital signs in the clinical setting, and in particular in the deteriorating patient. These devices as of yet have not been well validated in the clinical setting in the clinical ranges observed in a critically unwell patient. We will perform a systematic review of all novel wearable and contactless devices in the clinical setting with focus on degree of novelty and the range of vital signs captured.

**Methods:**

Ovid MEDLINE including Epub Ahead of Print and In-Process & Other Non-Indexed Citations, Ovid Embase, Cochrane Database of Systematic Reviews, Cochrane Central Register of Controlled Trials (CENTRAL) Health Technology Assessment (HTA) database (Ovid), CINAHL with Full Text, searches of the grey literature, cited references of eligible studies through Web of Science, and reference lists of eligible studies will be searched. Outcomes of interest will include the quality of studies in relation to reporting guidelines, limitations of non-invasive technology, and application in different clinical populations. We will perform a qualitative assessment of the *novelty* of the device and discuss its validation in deteriorating patients.

**Discussion:**

While novel monitoring devices are often proposed as a solution to problems with infection, discomfort, and frequency of monitoring in the clinical setting it has not yet been established which devices have been validated in clinical settings in the pathological ranges of vital signs that reflect patient deterioration. It is equally unclear what additional value these devices might provide. This systematic review will synthesize published data regarding devices that have been tested and validated in patients AND in a clinical setting AND in reference ranges that reflect severe illness.

**Systematic review registration:**

PROSPERO CRD42019130091

## Rationale

Minimally invasive patient monitoring has the potential to save lives and yet remains under-utilized and unproven. Identifying deteriorating patients in the clinical setting is important to minimize injury and promote faster recovery. An “unwell,” “deteriorating,” or “critically ill” patient has been described in a number of ways, but the commonly used convention for triggering an internal hospital alert for a deteriorating patient in Australia is the Medical Emergency Team (MET) call [1] which is commonly separated into 7 separate domains: a staff member subjectively worried about the patient, a heart rate (HR) less than 50 bpm or greater than 130 bpm, respiratory rate (RR) less than 8/min or greater than 30/min, a systolic blood pressure (SBP) less than 90 mmHg, oxygen saturations less than 90% on supplemental oxygen, a change in conscious state, or urine output < 50 ml/4 h. It has been shown that earlier intervention results in better outcomes and shorter lengths of stay in the hospital [2]. Delayed monitoring has been demonstrated to result in in-hospital cardiac arrests, resulting in increased morbidity to patients [3].

The rationale for better/novel monitoring therefore aims to bridge the gap between increased monitoring but reducing impact and disruption to patient care and ultimately move patient monitoring out of the clinical setting. Primary caregivers and patients both agree that less intrusive but continuous monitoring is likely to augment patient care [4] though innovation, including miniaturization of existing devices as well as alternative methods of measurement, will be required for this to be achievable.

The acceptable clinical standard for vital signs monitoring differs between institutions and even between clinicians within the same institution. For example, ward level monitoring of heart rate and respiratory rate has traditionally been performed by a bedside nurse via manual counting of the frequency [4] every 4‑8 h. Continuous monitoring of patient vital signs has been shown to improve times to response to deteriorating patients resulting in better outcomes. This likely results from less progression to cardiac arrest and more attendances by MET teams, [5–7]. Increased frequency of monitoring, however, can be equally associated with a number of detrimental effects to patients, namely injury/disease from the monitoring devices including infection risks, pain from intravenous or intraarterial monitoring devices [8], and skin barrier breakdown/pressure ulcers [9]. Furthermore, the reduced mobility associated with being tethered to a bed during monitoring can result in delirium, loss of sleep, or the need for admission to a high-dependency unit (HDU) which can paradoxically lengthen the length of stays in hospital [10] and cause long term damage to mental health [11].

The generally accepted clinical standard for heart rate monitoring using devices is the use of ECG monitoring [12] or pulse frequency from a photoplethysmogram [13]. Respiratory rate monitoring in patients requiring continuous monitoring is typically performed via thoracic bioimpedance from ECG leads or capnography [14]. Blood pressure monitoring in clinical spaces is typically performed via an automated oscillometric cuff [15], while oxygen saturation monitoring is performed using a two-wavelength pulse oximeter, either as a standalone device or integrated into a vital signs monitor [16]. Innovation with these devices over the past decades has been incremental rather than revolutionary. Despite advances in automation or miniaturization of parts, non-invasive blood pressure monitoring continues to rely on the same principle of collapse and reinflation of an artery then measurement by auscultation [17] or oscillemetry. Pulse oximetry, while now present in most smartphones [18] continues to rely on the same principle of the change in the absorbance of visible and infrared light of hemoglobin at different oxygen saturations [16]. Recent incremental innovations to other vital signs monitoring include new algorithms to better detect heart rate and respiratory rate [14] or the combination of multiple measurement devices into a single, more portable unit [19].

From an intellectual property perspective, a novel device is a device or concept not publicly available compared to existing prior art [20], with prior art being defined as the publically available information that attests to a device’s originality [21]. The general patentability of a novel device is subjective and specific criteria can vary from country to country [22], but must generally include an inventive step or new, non-obvious use [20]. Novelty and innovation when applied to diagnostic medical devices for general use in the clinical space suggests an improvement in the quality of the signal capture, a decrease in the resources needed to obtain the signal, increasing the physical locations in which the reading can take place, reduction in time taken to obtain the signal, or reduction in the price of the device [23].

Novel vital sign monitoring devices can be classified into either the wearable/contact or contactless space, with the former typically disposable sensors applied via adhesive [24], contained in a smaller device in direct contact with the body [25, 26] or in near or direct proximity to the patient via mattress or bedsheet using ballistography [27] or electrical bioimpedence [28]. Contactless devices can be divided into image and non-image based systems, with image-based systems typically employing RGB or infrared cameras, whereas non-image based systems have typically employed modalities including time of flight sensors [29, 30], acoustics [31], and radar [32, 33].

While many new devices make claims of the ability to monitor patients for illness [34], there appears to be very little literature documenting their effectiveness in the unwell, let alone patients that are deteriorating. Equally, it is unclear whether devices that have been proposed to monitor vital signs in non-traditional settings have ever been validated against gold standard measurements in a traditional setting, as a result, their effectiveness cannot be quantified. Even devices that utilize the same measurement method placed in a different location in the body may return very different results. For example, skin temperature is being measured on a peripheral location such as the forehead or ear may not reflect core temperature in the critically unwell [35]. Validation studies of infrared cameras at airports as one example during a flu epidemic showed extremely poor positive predictive values [36]. Extremes of vital signs, such as an HR > 140 or RR > 30, might show enough difference in character and intensity to be improperly interpreted by these novel measurement techniques. A recent meta-analysis and systematic review of novel continuous non-invasive blood pressure monitoring based on tonometry and volume clamp methods showed larger than acceptable inaccuracy and imprecision compared to gold standard measurements over normal physiological ranges of blood pressure [37]. Finally, the recent Apple Watch study [38], while showing great promise for measurement of tachycardias, measures one single aberrant rhythm, and not in the critically unwell.

A recent systematic review by Harford et al. [39] provides a thorough assessment of wireless video-based patient monitoring and highlighted several deficiencies with these devices, including (1) minimal testing or validation in the clinical setting, (2) testing predominantly in neonates, not children or adults, (3) insufficient data for validation in the laboratory setting, in particular questions over the time period tested, and the range of vital signs tested on healthy volunteers. Their review highlights which video-based modalities show the most promise for future monitoring systems. However, it omitted wearable devices, which while not as novel, have generated significant commercial and clinical interest especially in the community-based setting. A significant number of studies assessing vital signs parameters and comparing them to gold standard values in the sleep laboratory setting were equally omitted. Finally, while the clinical ranges over which the vital signs were measured were reported, there is no information about whether there was dose-dependent bias with these measurement modalities.

In addition to narrowing the scope to only include clinically relevant studies performed on patients, this review aims to widen the technological scope from video-based and contactless monitoring devices, to also include wearable devices including patches and smart textiles.

The primary aims of this systematic review therefore address the following questions:

1. Are there novel, minimally invasive, devices that have the potential to detect a deteriorating patient in the clinical setting?
2. Which, if any, have been validated in the clinical setting to measure vital signs, blood pressure, heart rate, respiratory rate, or oxygen saturations?
3. How are they novel and what added value do they provide over existing and traditional monitoring modalities?
4. Were they measured across a range that includes parameters that exist in a deteriorating patient?
5. How well do these devices correlate to a clinically acceptable standard? Is there dose-dependent bias in the readings? In particular, are the devices equally accurate in normal physiological range compared to extremes of physiology seen in deteriorating patients?

The secondary aim is to identify the quality of the evidence with respect to a modified Quality Appraisal of Reliability Studies (QAREL) scoring system [40].

## Methods/design

Methods of the systematic review have been developed in accordance with the Preferred Reporting Items for Systematic Reviews and Meta-Analyses (PRISMA) [41] guideline. The completed PRISMA-P [42] checklist is available as a supplementary file to this protocol (Additional file 1).

### Eligibility criteria

All papers that utilize a novel technology studied in a human population in a clinical setting and in an adult (age ≥ 18 years) population. Comparison of precision and accuracy to a clinically validated reference device will be included. Novelty for the purposes of this review is taken as a wearable or contactless device that utilizes a novel methodology to ascertain blood pressure, heart rate, oxygen saturations, or respiratory rate that is different than the clinical standard.

Devices that are limited to incremental improvements only and not substantially different in measurement modality or body location will be identified and differentiated from novel devices but not assessed or included in the final analysis. Such devices have already been validated across the range of vital signs. These “less novel” devices include, for example, a wireless form of an existing device used in exactly the same manner (e.g., a wireless sphygmomanometer) or an amalgamation of multiple devices using standard measurement techniques that have been condensed into a single unit (e.g., Philips SureSigns patient monitors).

Changes in urine output < 50 ml/4 h will not be studied due to the limited application of such a monitoring device and the absence of a clinical “gold standard” comparator. Changes in conscious state will be equally not studied as there is no current ward-based standard that is used as a routine. There are no limitations placed on the primary objective of this review as long as there is an existing gold standard monitoring technique. Studies which focused on a specific pathology but could feasibly have identified a vital sign were also included. For example, a device designed to identify atrial fibrillation (a specific type of fast heart rate) instead of heart rate.

We will include randomized control trials, cross-sectional studies, case series and reports, and pilot studies that compare any novel monitoring system to an established form of monitoring used in clinical practice. Authors of studies published in languages other than English will be contacted by email for assistance with data extraction. Studies taking place in a sleep lab will be included, as it is expected that the level of monitoring present in a sleep lab would be equal or greater than what would be expected in a clinical ward and some abnormal pathology would be expected to be present. Studies which utilized publicly available clinical data from databases such as the Medical Information Mart for Intensive Care (MIMIC) will also be included. As this study is interested in novel clinical monitoring and its ability to feasibly detect a deteriorating patient in hospital compared to established clinical standards, studies which took place in non-traditional settings with no routine monitoring, including nursing homes, the community, and outpatient clinics will be excluded. Studies which do not employ a clear reference standard will also be excluded.

### Information sources

Searches will be performed on Ovid MEDLINE including Epub Ahead of Print and In-Process & Other Non-Indexed Citations, Ovid Embase, Cochrane Database of Systematic Reviews, Cochrane Central Register of Controlled Trials (CENTRAL) Health Technology Assessment (HTA) database (Ovid), CINAHL with Full Text, searches of the grey literature, cited references of eligible studies through Web of Science, and reference lists of eligible studies.

While technical papers may present novel technology, it is unlikely that they would have been tested in a clinical population without being published in a clinical journal. Nevertheless we will include Institute of Electrical and Electronics Engineers (IEEE) Xplore Digital Library and ACM Digital library in our search. Abstracts from conferences will also be included.

### Search strategy

The search strategy will be guided by a qualified medical librarian. Initial terms of interest will encompass the expected technology used, the vital signs parameter being measured, and the clinical setting. Terms will be exploded where appropriate and MeSH (Medical Subject Headings) terms will be used. While it is expected that many of the papers will be recent publications from the preceding decade, all databases will be searched from database inception. Once searches are complete, duplicates will be removed. An example search strategy is included in Table 1.

**Table 1:** Example of search strategy

1	(wearable or patch or adhesive or fitbit or iwatch or smartwatch or jawbone).mp. [mp = ab, ti]
2	(hr or (heart adj1 rate) or pulse or pulse rate).mp. or actigraphy/is [mp = ab, ti]
3	((blood and pressure) or BP or perfusion).mp. [mp = ab, ti]
4	(oxygen saturation or SpO2 or oxygenation or saturation*).mp. [mp = ab, ti]
5	respiration or rr or breathing rate or respiratory or breathing mechanics).mp. [mp = ab, ti]
6	exp Vital Signs/ or vital.mp. [mp = ab, ti]
7	(contactless or cableless or contact sensor or unobtrusive or remote or wireless or non-contact or noncontact).mp. [mp = ab, ti]
8	(radar or ballisto* or accelerom*).mp. [mp = ab, ti]
9	(PPG or photople* or videopleth*).mp. [mp = ab, ti]
10	(thermography or infrared or thermal or thermograph*).mp.[mp = ab, ti]
11	(camera or RGB or image based or webcam or web-cam or video* or wavelet or ambient light or (optical and imag*) or camera-based or photoplethysmography).mp.[mp = ab, ti]
12	piezoelectric or impedence).mp. [mp = ab, ti]
13	clinical or PACU or intraoperative or intensive or ICU or ward or clinical or hospital or triage).mp. [mp = ab, ti]
14	(1 and 2) or (1 and 3) or (1 and 4) or (1 and 5) or (1 and 6)
15	7 and 8) or (7 and 9) or (7 and 10) or (7 and 11) or (7 and 12)
16	(15 and 2) or (15 and 3) or (15 and 4) or (15 and 5) or (15 and 6)
17	(14 and 13) or (16 and 13)
18	*Monitoring, Physiologic/ or monitoring.mp.[mp = ab, ti]
19	17 and 18
20	remove duplicates from 19

### Data management

The initial search strategy, references selected by title, and then abstract as well as full-text articles will be stored in the Covidence citation manager (Veritas Health Innovation, Melbourne AU). Data collected through this review will be individually collated into separate spreadsheets by each reviewer (NR, PC) then collated into a single datasheet.

### Selection process

Two reviewers (PC, NR) will appraise all full-text articles. In the event of a discrepancy, a third reviewer (IH) will resolve the difference.

Once all searches are performed and checked for duplicates, all titles and abstracts will be screened by two reviewers (PC, NR) and any unrelated articles removed. Any article where there is disagreement between the two reviewers will be included for abstract review. Any difference in opinion between the two reviewers will be included in the full-text review.

Full-text review will be extracted through the Covidence reference manager. During the review process of each article, the reference list will also be checked for any relevant articles not identified in the original search strategy. The reference list of relevant review articles will also be searched to identify potentially missed clinical studies.

Returned papers will then be classified into two major headings: contactless and contact (wearable) devices.

Each of these major headings will be further subdivided. It is anticipated that the contact devices will largely be divided into reusable wearable consumer technology and disposable device. It is anticipated noncontact devices will be divided into image and non-image-based devices.

Each of these subgroups will have additional classifiers added. We expect to stratify studies based on the vital sign measured and the population being studied.

### Data collection process

Two reviewers will work in tandem to collect data from each extracted paper. Information extracted from each publication will include: type and date of publication of the study, funding source, setting/patient demographic, number of participants, age of participants, body part(s) imaged or location of placement of wearable, comparison of device to a reference method and statistical test used for this comparison, type of reference method used, vital sign being studied, range (if applicable) over which the vital signs are studied, number of values (if identifiable) outside of MET call range (heart rate less than 50 or more than 130, systolic blood pressure below 90 mmHg, respiratory rate below 8 or greater than 30, oxygen saturation below 90%), documented illness severity, whether the patient was being actively or passively monitored, specification if each value is an individual value or an aggregate of multiple observations, number of false alarms reported, limitations reported, type of statistical test used, and main conclusions.

Devices will be scored for novelty and innovation using a modified PwC innovation classification scale [23] and the value creation matrix (Table 2). This score reflects the added value of the device and the degree of deviation from the current clinical monitoring standard. A simplified numeric score relating to the degree of innovation will also be applied—a score of 1 will be given to a device with the same use but a new algorithm, 2 to an incremental update to an existing device, 3 to a new and non-obvious use of an existing device or a substantial upgrade to an existing device, 4 to a radical change to an existing device or development of a completely new device and approach to measuring a vital sign (Table 3).

**Table 2:** Scoring criteria for level of device innovation; modified PwC value creation matrix

	Remove (−2)	Reduce (−1)	Retain (0)	Reform (+1)	Replace (+2)	Score
Value creation						
Quality of signal capture						
Quantity of resources needed for signal capture (nursing staff, doctor present, technician needed)						
Number of physical locations (clinical, nursing home) device can be used						
Time taken to obtain signal						
Price of obtaining signal						
Total score

**Table 3:** Scoring criteria for level of device innovation: innovation scale

Innovation Scale	
Criteria	Points
No appreciable innovation	0
Device with the exact same use but utilizing new algorithm	1
Incremental update to existing device	2
Non-obvious use of an existing device in new manner or a substantial upgrade to existing device	3
Radical change to an existing device or completely new device and approach to measuring vital sign	4

### Assessment of study quality

We plan on employing a tailored QAREL scale [40] (Additional file 2) to assess the quality of each diagnostic test study. Both reviewers (NR, PC) will independently score according to QAREL, with any discrepancies resolved by a third party (IH). These will then be separated by domain and summarized in either graphical or tabular format. Should specific domains of QAREL not be applicable to the study, this will be reported and presented as an “N/A” in the summary table.

Given the variability of the devices and the likely low patient acuity/risk cohort, it is unlikely that enough data will be available for quantitative analysis. Attempts will be made to summarize all existing statistical data from the Bland-Altman plots(B-A), should these be available. If no B-A plot is available, the alternative statistical test will be reported but no further analysis of these studies will be performed.

If the *x*-axis on the B-A plot is the mean of two measurements, the value of the gold standard will be calculated based on the mean and difference from each time point. To show whether the accuracy of the device is similar across all vital sign ranges, regression will be attempted on the B-A plot. If this is not possible with the available data, separate B-A plots or correlation calculations will be performed between datapoints in the normal and abnormal vital signs ranges. If no B-A plots are presented, the range of vital signs data will be estimated based on the demographics table.

If any of this information is not available on the scatter plots, we will attempt to contact the original authors. If obtaining enough primary data is not possible, the primary intended outcome will be a qualitative review of the studies which utilize novel wearable or remote monitoring devices with an emphasis on whether values exist outside MET call criteria and their relative performances compared to a gold standard as well as the degree of novelty. The studies will be categorized based on contact/contactless modality as well as the vital sign (HR, RR, BP, oxygen saturation) being measured.

### Amendments to protocol

Any deviation from this protocol will be dated and documented. No changes will be made to the main body of the protocol. Unanticipated additional findings will be discussed in the final systematic review.

## Discussion

Monitoring patients in the clinical setting can be costly, painful, time-consuming, inefficient, and inaccurate [8, 9]. While novel monitoring devices are often proposed as a solution to these problems, it has not been established which devices have been validated in patients at reference ranges that reflect clinical scenarios and patient deterioration. It is equally unclear what value these devices might provide. It is expected that this systematic review will synthesize which devices, if any, have been used in clinical settings, particularly in patients that might be clinically deteriorating.

The main strength of this study will be a summary of novel devices that have been tested in a clinical environment, and their usefulness in populations at risk of clinical deterioration. This will provide a basis on which monitoring modalities have potential future applications and provide more robust and adequately powered future research.

## Limitations

Despite an extremely broad search strategy encompassing many different measurement modalities, it is expected that very few studies will include patients that fall within the clinical range of the deteriorating patient category. While there is a possibility that some devices will not be included in the general literature due to intellectual property concerns, this also prevents clinicians and researchers from evaluating and validating the effectiveness of a commercial device in the critically ill. It has traditionally been difficult to measure the effectiveness of interventions in reducing rare but important events [43], and despite this review assessing the antecedent vital signs that might lead to the event rather than the event itself, there is still the possibility that the majority of devices would not have captured these vital signs.

There is also the high likelihood of publication bias, where studies that show a poor agreement between values established by the novel method and the “gold standard” would be not published. It is unlikely that unpublished studies would include devices useful in the clinical setting, though abstracts from conferences were included for the sake of completeness.

Of note, this review does not assess other vitals such as a change in conscious state or reduction in urine output due to the absence of validated standards. Future reviews should aim to include an assessment on devices that could assess wakefulness including video [44] wearable actigraphy monitors [45] to monitor consciousness. Similarly, monitoring of urine output [46] via weight or camera-based tools will not be included in this review. Any publications relating to the monitoring of these aspects of the deteriorating patient identified during this literature search will be noted for further analysis in a future review.

Finally, it should be noted that the absence of validation does not necessarily imply the absence of valid clinical application. For example, the American Society of Anaesthesiologists adopted the use of pulse oximetry as a standard in 1986 despite the absence of level 1 evidence of its effectiveness [16] at the time. Nonetheless this review will seek to provide further clarity on potential modalities that carry potential to transform patient monitoring.

## Supplementary information

**Additional file 1.** PRISMA-P Checklist

**Additional file 2.** Tailored QARELtool for quality assessment of diagnostic reliability

## Data Availability

The datasets used and/or analyzed during the current study are available from the corresponding author on reasonable request.

## Abbreviations

BP: Blood pressure
RR: Respiratory rate
B-A plot: Bland-Altman plot
MeSH: Medical Subject Headings
MET: Medical Emergency Team
CENTRAL: Cochrane Central Register of Controlled Trials
HTA: Health Technology Assessment database
CINAHL: Cumulative Index to Nursing and Allied Health Literature
