# Supplementary figures and images for: Novel wearable and contactless monitoring devices to identify deteriorating patients in the clinical setting: a systematic review protocol
Source: Syst Rev. 2020 May 6;9:104. doi: 10.1186/s13643-020-01370-1 (PMC7201723; doi:10.1186/s13643-020-01370-1)

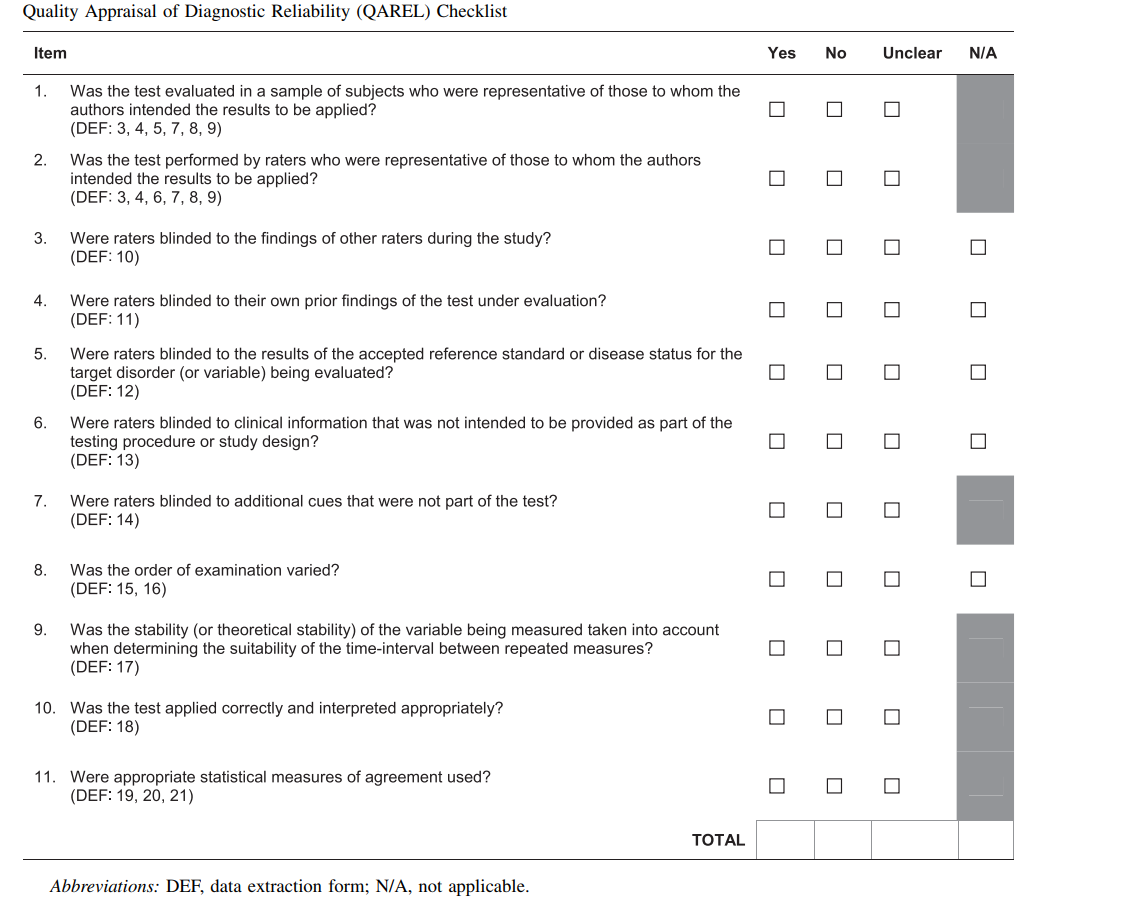

Supplement: Supplementary file 2 — Additional file 2. Tailored QARELtool for quality assessment of diagnostic reliability [file 13643_2020_1370_MOESM2_ESM.tif]
